# Engineered PLGA-PVP/VA based formulations to produce electro-drawn fast biodegradable microneedles for labile biomolecule delivery

**DOI:** 10.1007/s40204-020-00143-2

**Published:** 2020-11-03

**Authors:** Valentina Onesto, Concetta Di Natale, Martina Profeta, Paolo Antonio Netti, Raffaele Vecchione

**Affiliations:** 1 Center for Advanced Biomaterials for Health Care, CRIB, Istituto Italiano di Tecnologia, Largo Barsanti e Matteucci 53, 80125 Naples, Italy; 2 Interdisciplinary Research Center of Biomaterials, CRIB, University Federico II, P.leTecchio 80, 80125 Naples, Italy

**Keywords:** Electro-drawing, Microneedles, Transdermal drug delivery, Protein release, Controlled release, PLGA

## Abstract

Biodegradable polymer microneedles (MNs) are recognized as non-toxic, safe and stable systems for advanced drug delivery and cutaneous treatments, allowing a direct intradermal delivery and in some cases a controlled release. Most of the microneedles found in the literature are fabricated by micromolding, which is a multistep thus typically costly process. Due to industrial needs, mold-free methods represent a very intriguing approach in microneedle fabrication. Electro-drawing (ED) has been recently proposed as an alternative fast, mild temperature and one-step strategy to the mold-based techniques for the fabrication of poly(lactic-*co*-glycolic acid) (PLGA) biodegradable MNs. In this work, taking advantage of the flexibility of the ED technology, we engineered microneedle inner microstructure by acting on the water-in-oil (W/O) precursor emulsion formulation to tune drug release profile. Particularly, to promote a faster release of the active pharmaceutical ingredient, we substituted part of PLGA with poly(1-vinylpyrrolidone-*co*-vinyl acetate) (PVP/VA), as compared to the PLGA alone in the matrix material. Moreover, we introduced lecithin and maltose as emulsion stabilizers. Microneedle inner structural analysis as well as collagenase entrapment efficiency, release and activity of different emulsion formulations were compared to reach an interconnected porosity MN structure, aimed at providing an efficient protein release profile. Furthermore, MN mechanical properties were examined as well as its ability to pierce the stratum corneum on a pig skin model, while the drug diffusion from the MN body was monitored in an in vitro collagen-based dermal model at selected time points.

**Electronic supplementary material:**

The online version of this article (10.1007/s40204-020-00143-2) contains supplementary material, which is available to authorized users.

## Introduction

Biocompatible microneedles (MNs) have recently gained great attention, being recognized as a pain-free and successful administration route, alternative to injection that may cause pain, discomfort and avoidance by people with needle-phobia (Kim et al. 2012; Leone et al. 2017). MNs are employed for many applications ranging from vaccine delivery to biopharmaceuticals administration (Balmayor et al. 2011; Bariya et al. 2012). MN devices based on dissolvable or biodegradable polymers have been developed to overcome the drawbacks of solid microneedles, such as risks associated with tip breakages, low loading capability or difficulties in infusion systems (Lee et al. 2008; Park et al. 2005).

Most of the MN fabrication methodologies reported in literature, are based on micromolding (Jamaledin et al. 2020; Li et al. 2019; Nejad et al. 2018). Unfortunately, these mold-based techniques result in sequences of multi-step processes, temporally and economically expensive. In particular, hand solutions based on room temperature solvent casting are very time consuming (Bediz et al. 2014; Lee et al. 2008), while the use of UV treatment on photocurable polymers can inhibit the activity of the active compound encapsulated within the polymer matrix and the photoinitiator residuals within the final microneedles can introduce dangers in terms of toxicity (Sullivan et al. 2008).

Mold-free microneedle fabrication techniques, based on drawing lithography (Lee and Jung 2012), droplet-born air blowing (DAB) (Kim et al. 2013a, b), cyclic contact and drying process on pillars (CCDP process) (Kim et al. 2015), have been proposed as potential solutions to several of the technological limitations affecting micromolding. Nevertheless, these methods, despite being free of master fabrication processes or replica molding, are affected by limits arising from high process temperatures or features of reproducibility typically lower than the mold-based processes.

Among the mold-free techniques, electro-drawing (ED) has been recently proposed as a mild temperature, non-contact and UV-free process for the fabrication of poly(lactic-*co*-glycolic acid) (PLGA) polymer microneedles (Ruggiero et al. 2018; Vecchione et al. 2014). According to the ED method, microneedles are shaped in a single step starting from the deformation of drops of PLGA solution, by applying an electro-hydrodynamic (EHD) force on them.

Here, we enabled the use of ED microneedles to the encapsulation and the delivery of labile molecules such as collagenase by providing a faster degradable formulation as compared to PLGA. Indeed, as compared to the previous work, the focus of this work is on the formulation which provides a completely different microstructure, mainly based on a continuous porosity that guaranties drug release in a time-frame of few hours.

Collagenase is a zinc‐dependent metallo-proteinases able to degrade with a high specificity collagen substrates, playing a key role in the modulation of inflammation disorders, chronic wounds, ulcers or removal of cellular debris (Di Pasquale et al. 2019). The recent combination of collagen-lytic activity of collagenase with the wound healing properties of hyaluronic acid led to the production of a safe and active pharmaceutical formulation for the topical treatment of wounds, HYALO4 STARTMET (Di Pasquale et al. 2019). Unfortunately, being a protease, collagenase suffers of instability in physiological environment; in particular, it has been reported that it has a stability of maximum 8 h when it is stored at 4 °C or 24–48 months when storage temperatures range between − 20 and − 80 °C (Vaccaro et al. 2017). Starting from these considerations, our idea was to encapsulate it within a W/O single emulsion to have a polymeric shell as protection. Polymer-encapsulated drugs are in general more effective than their freely delivered counterparts, since polymer-loaded drugs are protected from degradation (McCall and Sirianni 2013). This protection provides a longer biological half-life and a potentially improved efficacy with reduced systemic side effects. However, once applied in vivo, proteins are subjected to a number of stability constraints due to the fact that being the delivery system in a wet condition, protein starts to be exposed to water even before being released (Wu and Jin 2008). For these reasons, the aim of this work is to shorten the prolonged release in a time window of few hours, reducing in this way the drug degradation over time (Kamaly et al. 2016). This has been possible since W/O emulsions can be easily controlled in their porosity degree during the fabrication process, which is a fundamental aspect in pharmaceutical formulations because the drug release strictly depends on the porosity of the material. Additionally, we optimized the emulsion formulation substituting part of PLGA with a copolymer poly(1-vinylpyrrolidone-*co*-vinyl acetate) (PVP/VA) as a continuous phase to accelerate polymer matrix dissolution. A relatively fast release is also useful to reach higher dosages as compared to slower release systems. For instance, in the case of collagenase, being it a labile molecule, low dosages may not achieve an in vivo therapeutic effect because of its serum inhibition (Badalamente and Hurst 2007).

Herein, different emulsion formulations were tested varying the PLGA-PVP/VA ratio as well as the amount and composition of the water phase. The results demonstrated that the addition of PVP/VA modifies the MN inner microstructure leading to the formation of interconnected porosity. The addition of maltose in the water phase helped in prolonging the W/O emulsion stability (Esposito et al. 2016). Microneedle morphology, inner structure and mechanical properties were also investigated. Collagenase entrapment efficiency, release and activity data were analyzed by fluorescence and UV confirming its functionality upon microneedle production process.

Moreover, MN ability to pierce the stratum corneum was confirmed on a pig skin model and the drug diffusion from their body was monitored in an in vitro dermal model at selected time points, such as 30 min, 2 h and 24 h.

Thanks to the ability in easily engineering the precursor emulsion formulation, our electro-drawn microneedles, could represent a new road in the pharmaceutical market of collagen-related pathologies.

## Materials and methods

### Materials

Collagenase (produced from Vibrio alginolyticusby Fidia Farmaceutici S.p.A.Noto, Siracusa, Italy, using a non-pathogenic aerobic bacterium (V. alginolyticus) by an aerobic fermentation process which produces a very purified enzyme lacking in microbial impurities (Vaccaro et al. 2017). Pyroelectric substrates were purchased from Altechna (LithiumTantalate LiTaO_3_ optical grade wafers, z-cut, 0.5 mm thick, double side polished).

Positive AZ®ECI 3012 photoresist and AZ 351 B developer were purchased from Microchemicals. 2-Propanol and acetone were both provided by Sigma-Aldrich. Titanium pellets (purity 99.95%) for metal deposition were purchased from KurtJ. Lesker Company, while wrapping wires and silver conductive paint employed for circuit connections were provided by RS Components. Polymethyl methacrylate (PMMA) sheets, with thickness ranging from 1 to 5 mm, were provided by Goodfellow. Further, Dow Corning Sylgard 184 Silicone elastomer, joint to its curing agent, was used for the fabrication of arrays of pillars and deposition vessels.

Poly(lactic-*co*-glycolic acid) with molecular weight in the range 38,000–54,000 Da (PLGA or RESOMER®RG 504 H), was purchased from Evonik Industries. Poly(1-vinylpyrrolidone-*co*-vinyl acetate) solution (molecular weight 13,000, 50 wt% in isopropanol) was purchased from Sigma-Aldrich. 24 h before usage isopropanol was left to evaporate and solidified PVP/VA was weighed as biopolymer for microneedles. Dimethyl carbonate (DMC, ≥ 99%) used as solvent for PLGA and d-( +)-maltose monohydrate (BioUltra, ≥ 99.0%, C.A.S. 6363-53-7) were purchased by Sigma-Aldrich. Egg lecithin (Lipoid E 80) was purchased from Lipoid (Ludwigshafen, Germany).

### Collagenase functionalization

Collagenase was covalently conjugated to Atto-488-maleimide using a previously reported procedure (Hermanson 2013). Briefly, a solution of 0.43 mg/mL of the enzyme (tris(hydroxymethyl)aminomethane **(**TRIS) buffer, pH 7.1) was mixed with a 20-fold molar excess of reactive dye for 1 h at 25 ^◦^C under magnetic stirring. Unbound dye was removed using a 3 kDa molecular weight cut-off Vivaspin 6 centrifuge filter. The correct enzyme-dye conjugation was validated by the UV–vis technique, as reported in our previous paper (Battisti et al. 2019).

### Collagenase-488 entrapment efficiency (%η) and release

The %*η* of collagenase-Atto 488 was measured by fluorescence as already reported using an EnSpire R Multimode Plate Reader and following the signal of functionalized collagenase at 520 nm (Battisti et al. 2019). To summarize, three patches of each configuration of microneedles were dissolved in a mixture of dimethyl sulphoxide (DMSO)/sodium dodecyl sulfate (SDS)/ sodium hydroxide (NaOH) for 30 min at room temperature. The quantification was performed using a titration curve of the free collagenase in the same conditions (Figure S1). As to the release, three patches of each configuration were suspended in 1.5 mL of TRIS buffer pH 7.1 and incubated at 37 ^°^C and at 500 rpm. From a time period of 30 min until 24 h, 1 mL of PBS was removed (at the equal time the patches were refreshed with the same amount of PBS and analyzed by fluorescence. Calibration curves of free enzymes were prepared in TRIS buffer (Figure S2).

### Collagenase activity

To validate the activity of enzyme inside microneedles, “collagenase chromophore substrate” assay was performed (Wuensch and Heidrich 1963). In particular, two patches of microneedles of each formulation were put in contact for 15 min with the collagenase chromophore substrate and the cleaved peptide was analyzed by UV–vis at 320 nm as described by Wuensch and Heidrich (1963). Data were normalized with respect to the amount of collagenase contained into each MN patch on the basis of the final amount of the obtained polymer.

### Integrated device fabrication

The fabrication of the integrated device on the pyroelectric crystal was carried out as described in (Ruggiero et al. 2018). Briefly, a thin layer of photoresist was deposited onto the crystal and spin coated (Laurell Technologies Corporation) for 60 s at 3000 rpm. To avoid electrical discharges due to the pyroelectricity, the soft-bake step was performed following a temperature ramp of 1 min and 30 s, from room temperature up to 90 °C. The sample was then gradually brought again to room temperature. Selective exposure was induced by a direct laser writer system (DWL 66 fs, Heidelberg Instruments Mikrotechnik) by setting, as exposure parameters, 60% for the beam intensity and 30% for the focus. The same thermal ramp of the soft-bake step was followed for the post-exposure bake treatment. The sample was then developed for 60 s and cleaned in deionized water. After the development, a titanium thin layer (having a thickness of 200 nm) was deposited onto the crystal using a thermal evaporator (Tecnoservice) equipped with a thickness controller (Inficon). Then, a lift off process of 15 min was carried out by immersing the sample in pure acetone within a sonicating bath, to obtain the final desired metallic pattern. Finally, the sample was cleaned and dried with nitrogen. Titanium thickness was measured by performing a profiler analysis (VeecoDektak 150). The fabricated device was then finalized by welding connection wires.

### Fabrication of the arrays of pillars and of deposition vessels

Polydimethylsiloxane (PDMS) arrays of 4 × 4 cylindrical pillars characterized by a center-center distance of 600 µm, diameter of 300 µm and height of 300 µm, were fabricated by replica molding onto a poly(methyl methacrylate) (PMMA) substrate. PDMS was chosen as an example of flexible support to show the ability to directly obtain microneedles onto a flexible substrate which is a potential advantage as compared to other manufacturing techniques where the microneedles are connected to each other and therefore they cannot be thought for a conformal application of the patch on the skin.

**Fig. 1:**
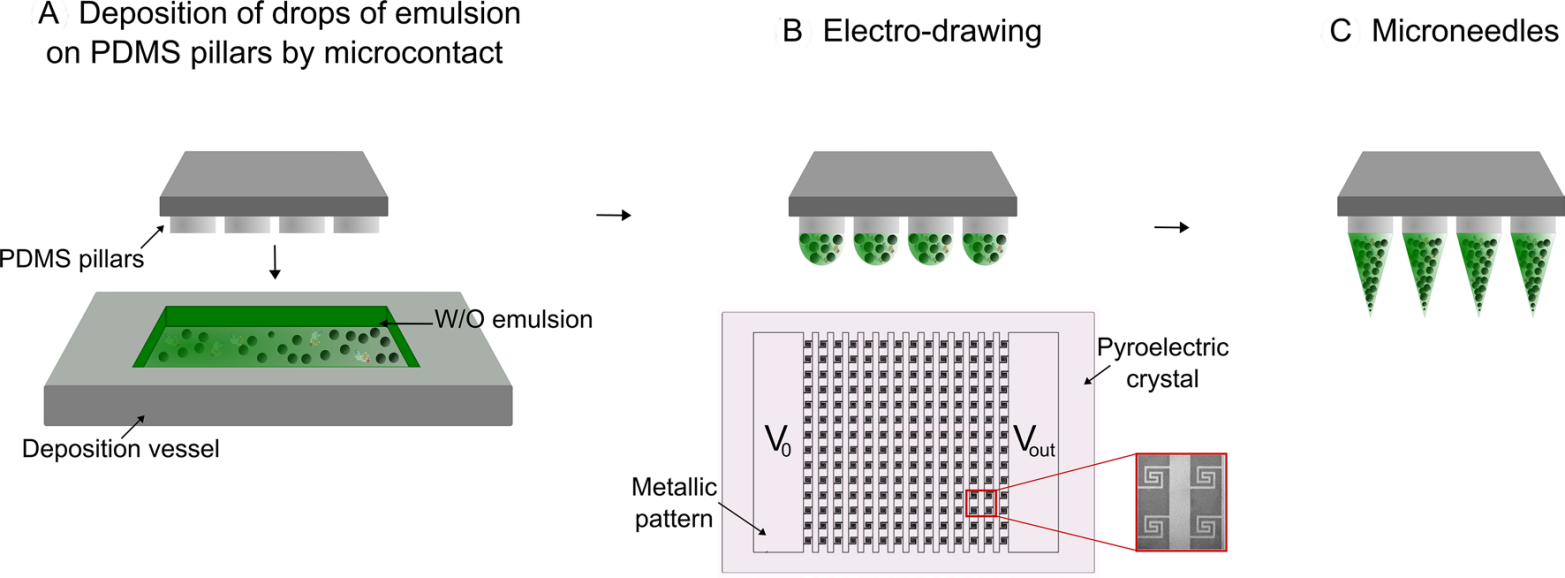
**a** Water-in-oil emulsion containing collagenase in the water phase is deposited within a vessel. Then, PDMS pillars, moved by motorized stages, come in contact with the emulsion forming drops. **b** A voltage is applied to the titanium circuitry patterned on the pyroelectric crystal and **c** microneedles are shaped by the action of the uniform electric field

Square PDMS vessels, used as tanks to deposit the emulsion (Fig. 1a), with edge of 1 cm and depth of 3 mm were obtained with the same method.

Briefly, PMMA molds reproducing the negative of the desired geometries (of the pillars or of the vessel) were fabricated by micromachining (Minitech CNC Mini-Mill). The obtained PMMA master was then cleaned from milling process debris by sonication in water for 10 min and dried with compressed air. PDMS elastomer was mixed with the curing agent in the 10:1 w/w ratio. Air bubbles arising from the mixing process were removed by putting the mixture in a vacuum chamber for 30 min. The PMMA master was filled with the mixture, which was degassed again for further 20 min. A 45 min thermal treatment at 80 °C was used to cure the PDMS. The PDMS was finally peeled off from the PMMA master.

### Preparation of the emulsion

PLGA and/or PVP/VA were dissolved in DMC as a non-toxic and environmentally friendly solvent, in percentages ranging from 20 to 40% w/v (Table1). This represents the continuous phase of the W/O emulsions. Two different water phases were tested in our emulsions. In particular, the first set of samples (AB) were produced with ammonium bicarbonate (7.5% w/w respect to polymer) dissolved in TRIS. In the other set of samples (LM), 180 mg/mL of lecithin were dissolved in TRIS; maltose was subsequently added until saturation (corresponding to 360 mg/mL). In both the water phases, collagenase-Atto 488 was added in a concentration of 188 µg/mL. The water phase was added to the oil phase in different amounts (Table 1) and emulsified using an immersion emulsifier (Ultra-turrax, IKA T-25 ULTRA-TURRAX Digital High-Speed Homogenizer Systems) for 1 min at 15,000 rpm, keeping samples in a nice bath to preserve protein activity.

**Table 1:** Different water-in-oil emulsion formulations employed to fabricate microneedles by electro-drawing and corresponding collagenase entrapment efficiencies and releases after 3 h

Case	Oil phase	Water phase (with collagenase)	Entrapment%	Release after 3 h %
c1	PLGA 200 mg in 1 mL DMC	200 µL AB	100	2
c2	PLGA 200 mg in 1 mL DMC	200 µL LM + AB	89.9	7.4
c3	PLGA 200 mg in 1 mL DMC	200 µL LM	74.5	6.5
c4	(PLGA 100 mg + PVP/VA 100 mg) in 1 mL DMC	200 µL AB	100	3.2
c5	(PLGA 100 mg + PVP/VA 100 mg) in 1 mL DMC	200 µL LM + AB	59.7	4.9
c6	(PLGA 100 mg + PVP/VA 100 mg) in 1 mL DMC	200 µL LM	67.2	10.4
c7	(PLGA 50 mg + PVP/VA 150 mg) in 1 mL DMC	200 µL LM	69	10.1
c8	PVP/VA 200 mg in 1 mL DMC	200 µL LM	43.6	20.6
c9	(PLGA 50 mg + PVP/VA 150 mg) in 1 mL DMC	250 µL LM	48.18	27.1
c10	(PLGA 50 mg + PVP/VA 150 mg) in 1 mL DMC	200 µL LM	68.19	13.35
c11	(PLGA 50 mg + PVP/VA 150 mg) in 1 mL DMC	350 µL LM	59.3	23.8
c12	(PLGA 50 mg + PVP/VA 150 mg) in 1 mL DMC	500 µL LM	72.8	24.3
c13	PVP/VA 400 mg in 1 mL DMC	250 µL LM	57.3	26.9
c14	PVP/VA 300 mg in 1 mL DMC	250 µL LM	56.1	21.9

### Fabrication of the microneedles

A volume of 0.25 mL of polymer emulsion was deposited by syringe within the PDMS vessel. To obtain controlled volume drops (0.01 μL) of emulsion on the top of the pillars, the PDMS pillars in upside-down position on a motorized stage (Thorlabs) were positioned 1 mm down the top of the vessel, coming in contact with the emulsion (Fig. 1a). Pillars are then brought, through the motorized stages, to a distance from the pyroelectric crystal equal to the desired final microneedle length and the electro-drawing of drops in microneedles was obtained by the action of the uniform electric field generated from the pyroelectric effect, upon the voltage was applied to the circuitry (Fig. 1b and c). The evolution of the microneedle shape was monitored by a lateral camera (Dino Lite). The microneedle shape was consolidated for 5 min (for PLGA MNs) or 10 min (for MNs containing PVP/VA) under the action of the electric field, after having increased the distance of 1 cm from the previous position. To be sure of the storage conditions, we kept our microneedles in boxes containing de-drying agents.

### Morphological analysis of MN inner microstructure

To investigate chromophore distribution inside the MNs, MNs were analyzed by confocal microscopy (Leica Microsystems TCS SP5 II, Germany) at 488 nm using a 20 × microscope objective. All images were acquired with a resolution of 1024 × 1024 pixels. Sections of matrices were prepared using Leica CryoUltra Microtome EM-FC7-UC7. Thin strips were incorporated in polydimethylsiloxane (PDMS), cured for 24 h at room temperature, and then frozen at − 140 °C. Finally, they were sectioned at a thickness of 5 μm. Pictures of porosity were taken using field emission SEM (Ultra plus Zeiss, Jena, Germany imposing 5 kV of voltage (EHT), after sputter coating of the samples with a 20 nm thick gold layer.

Acquired SEM images of MN sections were analyzed using ImageJ (V. 1.52p, Wayne Rasband, National Institutes of Health, Bethesda, MD,USA) to conduct a semi-quantitative analysis of pore sizes and porosity percentages.

Pores were identified selecting regions of interest of the SEM images (Figure S3) and, subsequently, the areas of all pores were summed and related to the total area of the considered image, according to Eq. 1:

1 $${\text{Porosity}}\,\, \left( \% \right) = \frac{{\sum A_{{{\text{pore}}}} }}{{A_{{{\text{tot}}}} }}100$$

where $A_{{\text{pore }}}$ is the value of the area of each pore, and $A_{{{\text{tot}}}}$ is the total area of the acquired image. To quantify porosity (%), 9 slices for each different type of MN formulation (three different slices belonging to three different samples) were considered.

### Mechanical failure test

To measure the force a microneedle can withstand before mechanical failure under an axial load, a displacement force test station (Instron 5943L4717), equipped with two metallic plates was used. A compressive method was created and load vs displacement curves were generated by measuring force and displacement at a rate of 0.1 mm/s. Images of the microneedle before and after the compressive test were acquired using a stereomicroscope (Olympus SZ61) to determine the mode of failure.

### Microneedle insertion assessment into Cadaver pig skin

To determine microneedle ability to pierce the stratum corneum, these were inserted into porcine cadaver skin. Single MN having height 600 µm and base 300 µm was electro-drawn on a PMMA cylindrical pillar. PMMA pillars were chosen to test the MN capability of piercing the stratum corneum, independently from its support, having PMMA a higher elastic modulus. The pillar was fabricated employing a micromilling machine (Minitech CNC Mini-Mill) and it had a diameter of 300 µm and a height of 300 µm.

Excised porcine skin was shaved using a razor and the skin’s subcutaneous fat was removed using a scalpel. The processed skin was cut into pieces and taken overnight in a phosphate buffered saline/ penicillin–streptomycin (PBS/PS) (1:1 v/v) solution at 4 °C.

The surface of the skin was dried with laboratory filter papers (Whatman®) and the microneedles were inserted by thumb into the skin for 3 min. After that, microneedles were gently removed from the skin. Both the microneedles and the porcine skin were imaged by means of a stereomicroscope (Leica EM UC 7) before and after the insertion assessment. Moreover, a hydrophobic dye, the methylene blue solution (MB) (Sigma-Aldrich), was placed on the skin for 1 h to stain the microchannels created from the microneedles penetration. The skin was then washed thoroughly in PBS to remove the excess dye from the surface. Finally, the skin was again imaged using a stereomicroscope to identify the hole left by the microneedle insertion (Nguyen et al. 2018) and the image of a MB-treated piece of skin was also taken as negative control.

### In vitro dermis equivalent: preparation and characterization

The 3D in vitro dermis equivalent model was set up embedding human dermal fibroblast (HDF) cells in a collagen matrix. Cells were cultured with complete medium, composed of Eagle’s minimal essential medium (EMEM) supplemented with 20% FBS, 1% glutamine, 1% non-essential amminoacids and 100 U/mL penicillin, 100 mg/mL streptomycin. The cells were maintained in 100 mm diameter cell culture dishes in a humidified controlled atmosphere at 37 ºC and 5% CO_2_. The medium was changed every 2–3 days. Collagen gels were prepared using rat-tail collagen solution (ibidi®) and reconstituted according to the manufacturer’s protocol. HDF cells of 6 × 10^4^ were suspended in 300 µL of 4 mg/mL collagen solution and poured into a Millicell® (Cell culture inserts, Millipore). The system was then incubated at 37 °C for 30 min to allow collagen fibrillogenesis and, afterwards, fresh cell culture medium was added to the gel. Finally, cells were incubated with DRAQ5 (Abcam) diluted 1:1000 in PBS for 30 min at RT for cell nuclei staining, to characterize the model by means of confocal microscopy (Leica Microsystems TCS SP5 II, Germany). Images were acquired with a 25 × water immersion objective and a resolution of 1024 × 1024 pixels.

### Release test into the in vitro dermis equivalent model

To determine microneedles ability to penetrate into the dermis and release their cargo, microneedles were inserted into the collagen model and the fluorescent signal of the encapsulated enzyme was followed at different time points (i.e. 30 min, 2 h, and 24 h). The results were obtained by confocal microscopy (Leica Microsystems TCS SP5 II, Germany) with a 10 × water immersion objective and images were acquired with a resolution of 1024 × 1024 pixels.

## Results

### Fabrication of microneedles and analysis of their inner microstructure.

**Fig. 2:**
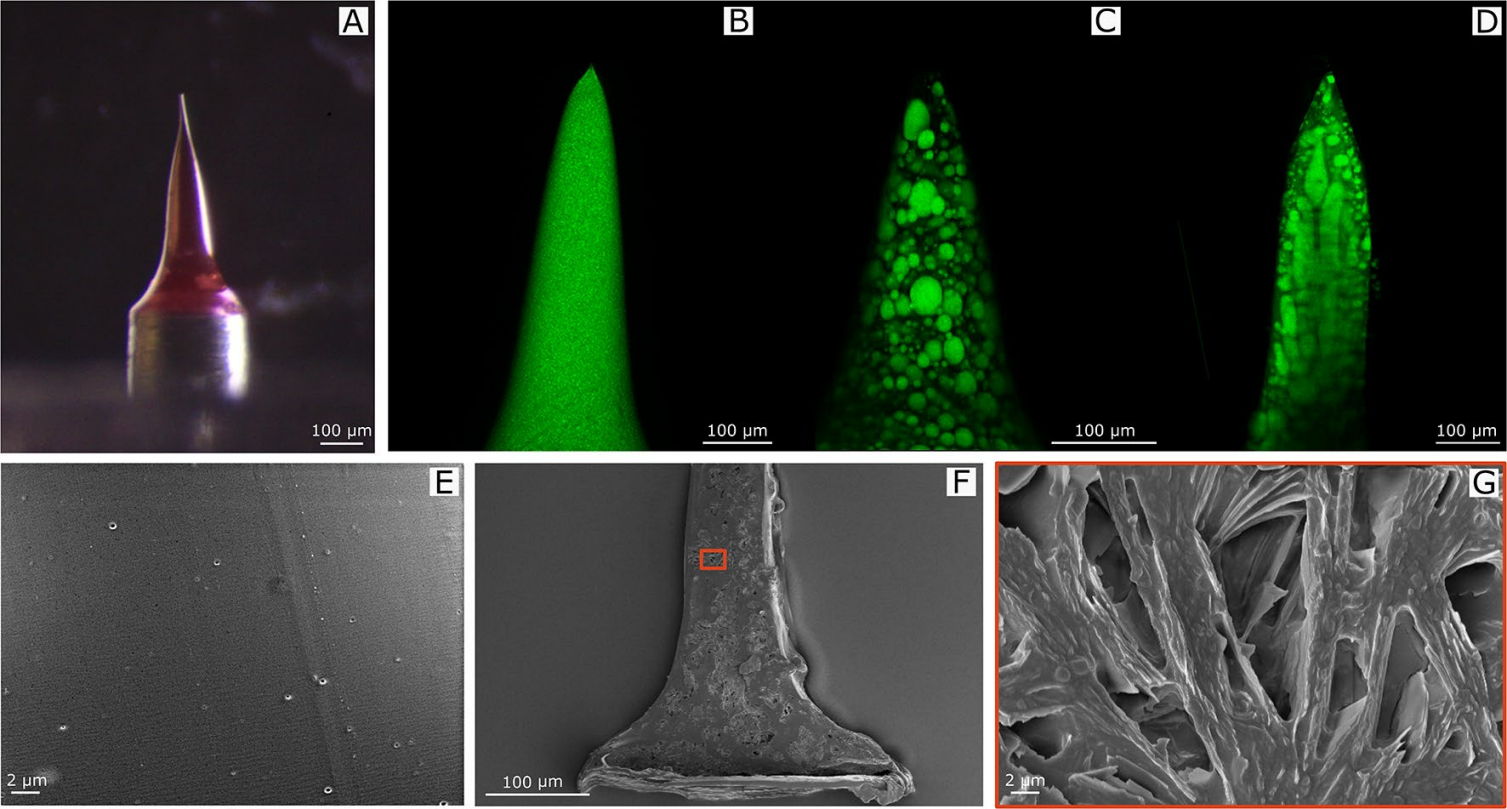
**a** Optical image of an electro-drawn microneedle made of PVP/VA + PLGA. Confocal microscopy images of collagenase-488 entrapped in electro-drawn MNs made of **b** 200 mg PLGA in 1 mL DMC with 200 μL of water solution ofammonium bicarbonate (7.5% w/w respect to polymer) dissolved in TRIS, **c** 200 mg PLGA in 1 mL DMC with 200 μL of water solution containing lecithin (180 mg/mL in TRIS) and maltose (360 mg/mL in TRIS), **d** 150 mg PVP/VA + 50 mg PLGA in 1 mL DMC with 250 μL of water solution containing lecithin (180 mg/mL in TRIS) and maltose (360 mg/mL in TRIS). SEM microscopy of MN longitudinal sections showing **e** the closed porosity of PLGA with ammonium bicarbonate MNs and **f**, **g** the interconnected structures of PVP/VA + PLGA with lecithin and maltose MNs

We tested different emulsion formulations, finalized to the formation of microneedles by the electro-drawing technique. Electro-drawing is a mask-less and mold-less 3D lithography process by which microneedles are fabricated under the action of electro-hydrodynamic pressure induced by a pyroelectric effect (Ruggiero et al. 2018; Vecchione et al. 2014), Fig. 1b and c. An optical image of a PVP/VA + PLGA electro-drawn microneedle morphology is shown in Fig. 2a**.**

Microneedle porosity represents a big issue in drug delivery applications since it is related to the amount of drug that can be loaded inside the polymeric matrix, and as a consequence, successively released in the body. With the electro-drawing technology, the microneedle inner microstructure can be flexibly and easily engineered playing with the precursor water-in-oil formulation, to tune drug release profile. In particular, to obtain a fast protein release kinetics, we studied 14 different emulsion formulations, analyzing consequent MN porous structure as well as the entrapment efficiency, release profiles and activity of collagenase (Table 1). We started from emulsions containing PLGA polymer 50: 50 (20% w/v) dissolved in 1 mL of dimethyl carbonate (DMC) and 200 µL of collagenase-488with the addition of 7.5% of ammonium bicarbonate (Table 1, case c1), which was previously used for the fabrication of porous microparticles for drug delivery (Di Natale et al. 2020). Needle microstructures were observed by confocal microscopy and ammonium bicarbonate showed to produce low size pores, with diameters of 1.5 ± 0.6 μm (Fig. 2b**,** Figure S3). Therefore, the water solution of ammonium bicarbonate was replaced by 200 µL of d-maltose monohydrate and lecithin, dissolved in the protein buffer (TRIS buffer, pH 7.1). This solution allowed the formation of internal microstructures expressing higher pore sizes of mean diameter of 15 ± 2.7 μm (Fig. 2c). Moreover, the presence of maltose limited the phenomena of coalescence and phase separation and the emulsion was more stable over time.

Once defined the water part of the emulsion, we started to study the nature of the polymeric matrix. Because the PLGA used for our study degrades in vivo in a couple of weeks (Ma et al. 2011), we combined it with poly(1-vinylpyrrolidone-co-vinyl acetate) copolymer in different ratios (Table 1). As we can see from Fig. 2d, emulsions with PVP/VA in the oil phase and lecithin and maltose in the water phase, with the formation of microneedles with interconnected pores, evenly distributed throughout the length of the cone. Different types of PVP are reported to be able to produce high-porosity structures, such as cross-linked PVP, homopolymers (with different molecular weights) or copolymers (Malik et al. 2019). Likewise, an enhancement of porosity into biomedical formulations has been reported using PVP as additive (Franco and De Marco 2020). Moreover, replacing some of the hygroscopic VP repeat units with hydrophobic repeat units of VA as is the case of PVP/VA copolymers (PVP/VA) a greater physical stability of biomedical structures can be obtained (Prudic et al. 2014).

The inner microstructure was even characterized by SEM microscopy of longitudinal sections, indicating confocal microscopy observations and an evidence of the interconnected pores, which represents an improvement as compared to the starting formulation case (Fig. 2e–g). Moreover, the porosity percentage, expressed as the sum of the areas of all pores with respect to the total area (Methods 2.9), was of 41% against the 0.4% of the starting formulation (Figure S3).

Final considerations are related to ED fabrication and pre-fabrication aspects of the MNs. In general, W/O emulsions containing PVP/VA in the continuous phase needed, as a microneedle was just electro-drawn, a 10 min shape consolidation time under the action of the electric field, higher compared to the 5 min of the sole PLGA, but still suitable considering that ED is a single step fabrication process. In some cases (c8) emulsion density was very low preventing the microneedle shaping through electric field application. when PLGA was totally absent (c13, c14), MN formation was possible but it was difficult to control the shape. Even if water phase densification by the addition of maltose limited phenomena of coalescence and phase separation, in other cases (c11, c12) the typical water-in-oil (W/O) emulsion instability was encountered: after 15 min we tested stratification of water phase in the upper part of the emulsions, because of the very high water phase amount in these formulations, tested to increase the release of the protein.

### Characterization of the entrapment of collagenase in the microneedles as a consequence of MN inner microstructure

**Fig. 3:**
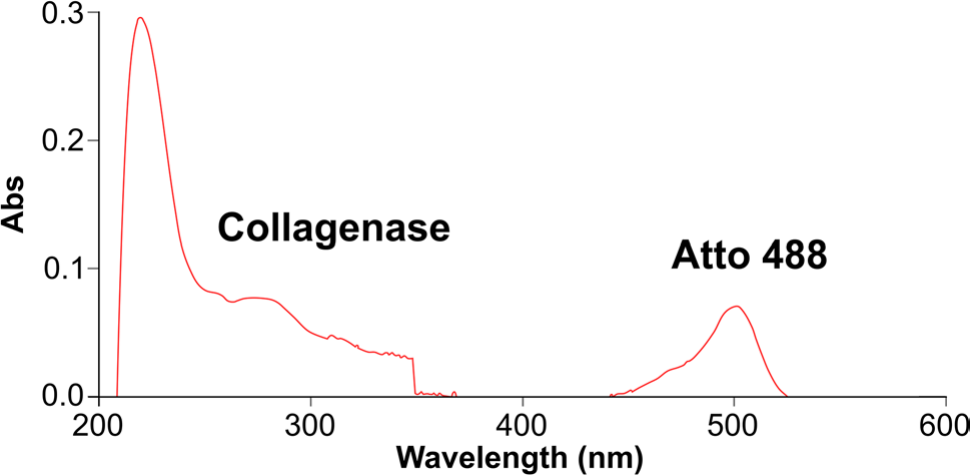
UV–vis of collagenase-Atto 488 functionalization

To evaluate the content of the enzyme into all formulations, we conjugated the ATTO 488 to the cysteine side chain of the protein, as reported in the Materials and Methods. The correct conjugation was evaluated by UV–vis recording the spectrum of the enzyme after the functionalization and purification reactions. Figure 3 confirmed that reaction was occurred, showing two peaks: one at 500 nm that is typical of the dye and the other at 280 nm associated to the protein. Afterward, using fluorescence (Figure S4), we calculated the %η of the enzyme for each formulation. The results reported in Table 1, showed that the encapsulation efficiency of collagenase-488 in the microneedles was major of 40% for all compositions, reaching 100% for c1. This result was expected as in electro-drawing the emulsion containing the collagenase is directly shaped to form microneedles limiting the protein loss. Nevertheless, the addition of agents such as lecithin and maltose, seems to reduce the encapsulation efficiency which is probably due to a decrease in the pH of the emulsions (calculated to be equal to 5.01) becoming closer to the pH of the protein (4.5) with possible generation of protein aggregation phenomena.

### Characterization of the in vitro release of collagenase loaded microneedles

**Fig. 4:**
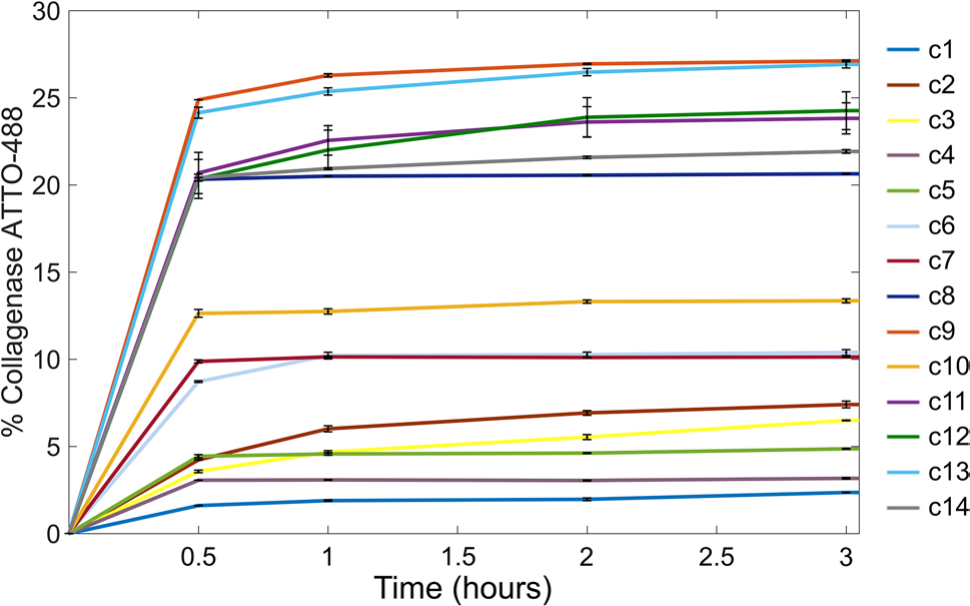
In vitro release of collagenase-Atto 488 from different microneedle formulations, in TRIS buffer pH 7.1. Time periods from 0 to 24 h

In vitro release profiles of microneedles containing collagenase were performed as reported in the Materials and Methods section. As shown in Fig. 4, after 24 h, PLGA 50 mg + PVP/VA 150 mg + LM (c9) and PVP/VA 400 mg + LM (c13) formulations released 27% of the enzyme, producing the most efficient enzyme diffusions. Contrary, the less efficient enzyme releases were obtained from PLGA with AB/AB + LM (c1, c2, c3) or from PLGA-PVP/VA with AB/AB + LM (c4, c5) using a minor amount of polymer or equivalent amount of PLGA/PVPVA. These data are coherent with confocal and SEM characterization of the microneedles which revealed the formation of internal microstructures with high porosity for formulations where LM were added. Moreover, the increase of the amount of PVP/PVA in the oil phase led faster collagenase release, confirming the interconnected porosity and the upgrading of the starting formulation case. Moreover, all experiments showed a plateau level at about 3 h and this is due to the fact that the release becomes so slow that collagenase degrades before accumulating at a critical detectable concentration. This consideration was corroborated by the data obtained incubating collagenase at 37 °C at 3 different concentrations (22 µg/mL, 0.22 µg/mL and 0.022 µg/mL) and for different time periods (from 0 to 4 h). As shown in Figure S5 A-B, protein fluorescent signal started to decrease after 3 h due to the exposure over time to the aqueous environment which initiates degradation phenomena.

### Characterization of the activity of collagenase in the microneedles

To validate the activity of the enzyme inside the microneedles, we selected the three best formulations derived by the entrapment and in vitro release results (c3, c9, c13). Using the assay reported in the Materials and Method section, three patches of the designated formulations were dissolved in TRIS buffer and incubated at 37 °C for 15 min together with the synthetic collagenase substrate. The peptide cleaved after this time period was analyzed by UV–vis following the signal at 320 nm (Figure S6- c3_a, c3_b, c9_a, c9_b, c13_a and c13_b). Empty microneedles and standard collagenase solution were used as negative and positive controls, respectively (Figure S6- blank and STD). As revealed in Table 2, the c3 formulation showed the highest percentage of the activity (70.8%), while the c9 and c13 displayed only 37.8% and the 18.3% respectively. Even if for formulation c3 collagenase stored a high biological activity after the MN production process, due to the low porosity of the MN structure, the protein remains embedded inside the PLGA phase and this allows a release of only 6.5% after 3 h. On the contrary, c13 maintains good release and entrapment efficiency, but it shows the lowest biological activity. Moreover, as discussed above, it was difficult to control the MN shape during the electro-drawing of PLGA-free (c13, c14) formulations. Lastly, case c9 shows intermediate percentages of activity as well as of entrapment and the highest collagenase release (27%) after 3 h, because of the interconnected porosity of the inner microstructure. For these reasons, c9 was chosen as the most suitable emulsion preparation for electro-drawn MNs.

**Table 2:** Activity percentage of collagenase inside microneedles

Formulation name	Activity %
c3	70.8 ± 0.4
c9	37.8 ± 7.1
c13	18.3 ± 1.8

### Mechanical failure test

**Fig. 5:**
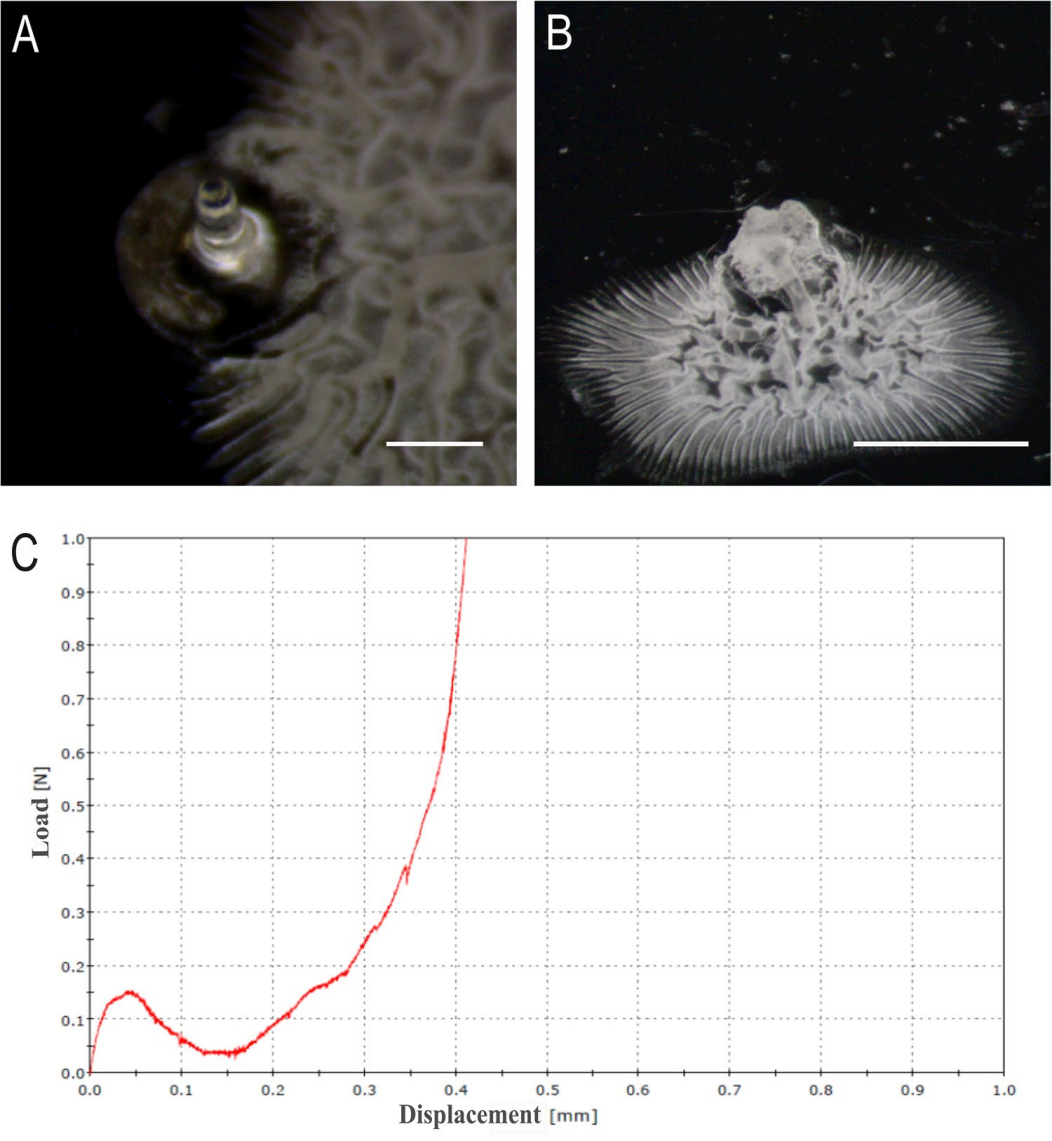
Light micrograph of a single microneedle (height 600 µm; base 300 µm) before (**a**) and after (**b**) an axial failure test; scale bar 300 µm. **c** Failure behavior of the microneedle under an axial load. Needle failure is identified by a sudden drop in force

The force required to cause needle failure by axial loading was measured to determine if microneedles are strong enough to pierce the skin without breaking. Data show (Fig. 5c) that upon needle failure, the force suddenly fall and the maximum force applied immediately before the decline was interpreted as the force of needle failure (0.123 N/needle). These results are in accordance with the work by Park et al. where their microneedles are able to indent the skin with an insertion force over a range of 0.10–0.22 N/needle (Park et al. 2006, 2005).

Moreover, as shown in Fig. 5a and b, the single needle was examined using a stereomicroscope before and after the failure test to show the deformation due to the compressive test.

### Microneedle insertion assessment into Cadaver pig skin

To test the microneedle ability to pierce the stratum corneum, single MNs on PMMA cylindrical pillars were prepared and inserted by thumb into a piece of cadaver pig skin and then removed after 3 min. Images of the skin before and after the needle insertion were acquired. After that, the MB-solution was applied for 1 h on the pierced skin. MB is a hydrophobic dye able to diffuse into the disrupted areas of the skin but not into the intact and lipophilic vicinity (Nguyen et al. 2018). Also in this case the skin was examined by light microscopy to image the piercing site.

**Fig. 6:**
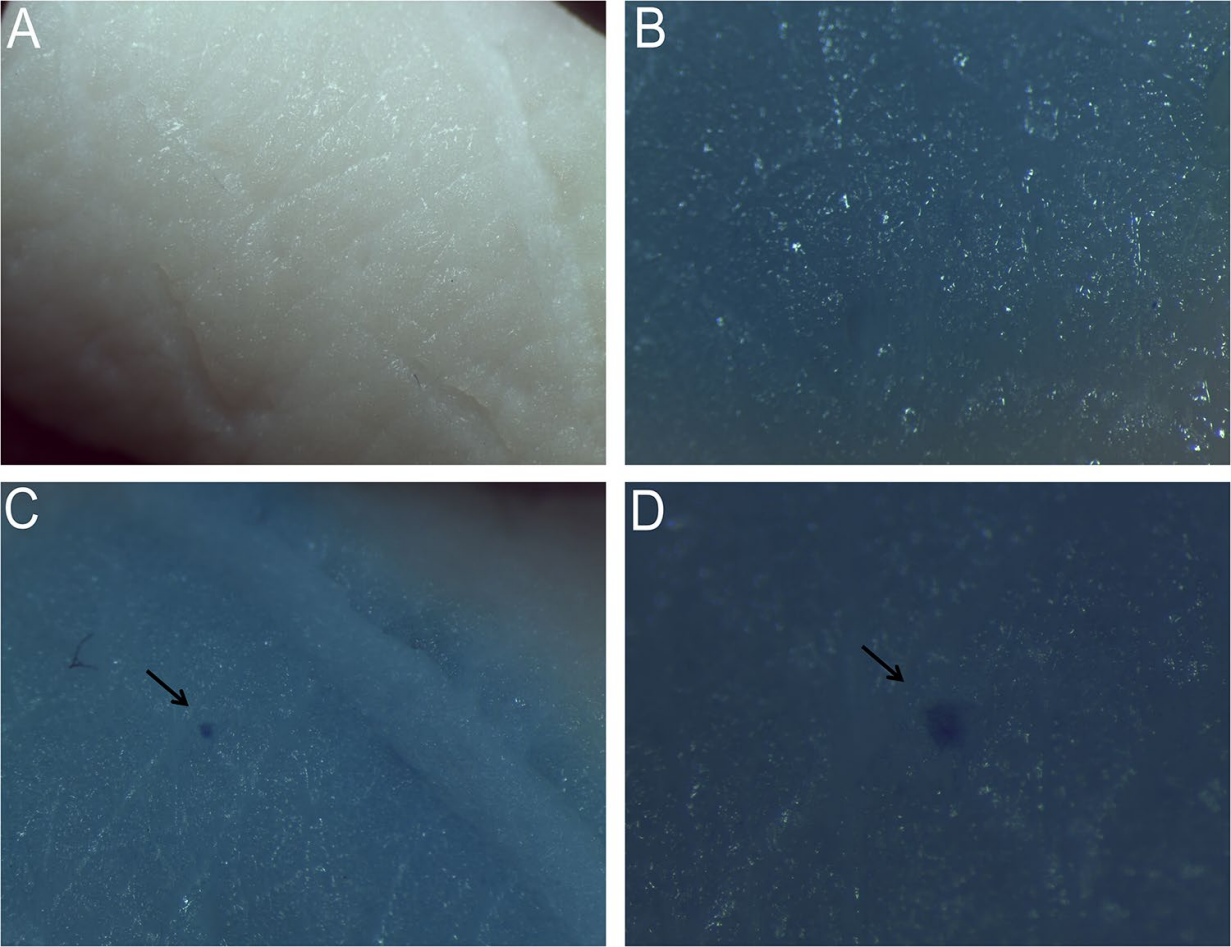
Microneedle insertion assessment into Cadaver pig skin. **a** Skin before insertion, **b** MB-treated skin with no insertion, **c** and **d** MB-treated skin after insertion

Image Fig. 6 contains representative optical photomicrographs, which show the skin before (Fig. 6a) and after indentation (Fig. 6c, d) stained with MB and an untreated piece of skin stained with MB as negative control (Fig. 6b). In particular, images Fig. 6c, d show the resulting blue dot on the epidermis. This indicates that the polymer microneedle was successfully inserted into skin piercing the stratum corneum.

### Microneedle release test into the in vitro dermis equivalent

**Fig. 7:**
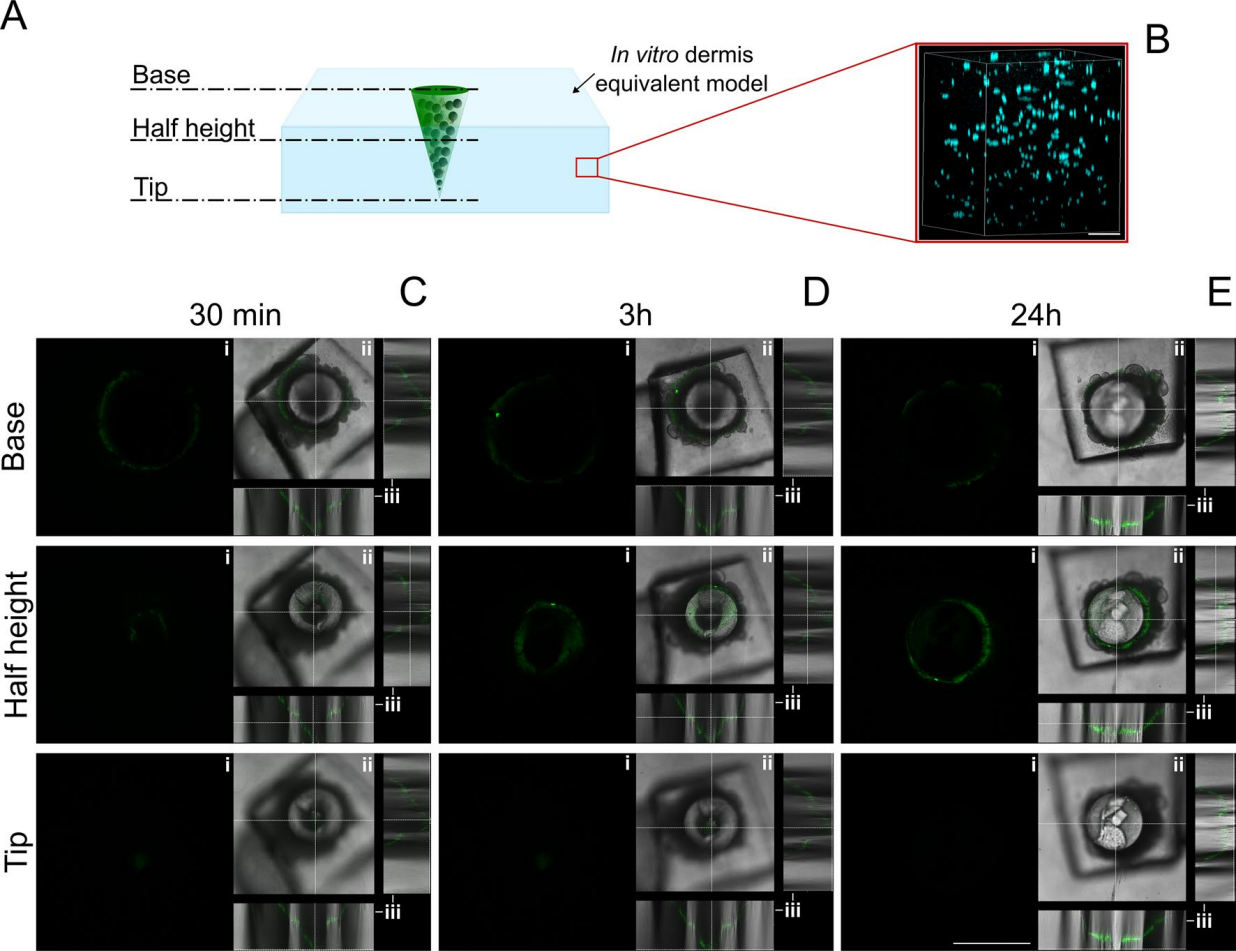
**a** Images were acquired by confocal microscopy at z-positions corresponding to the base, the middle height and the tip of the microneedle. **b** 3D reconstruction of the in vitro skin equivalent by means of confocal microscopy; cells nuclei can be seen inlight blue. Scale bar 100 μm. **c** Release test of the microneedle in the in vitro skin equivalent model after 30 min, 3 h, 24 h (top, middle and bottom views). Scale bar 600 μm

The in vitro dermis equivalent model was characterized by means of confocal microscopy. In particular, cell nuclei were stained and a 3D reconstruction of 2D confocal image stacks was recreated (Fig. 7b). The image shows that the cells are uniformly distributed throughout the collagen volume.

To test the collagenase release from a MN inside the in vitro dermis model, a microneedle was indented in the collagen and the enzyme fluorescence (i.e. the green signal) was followed overtime by means of confocal microscopy. In particular, three sampling times were chosen: 30 min, 2 h and 24 h. At each time point the images of three slices of the MN (i.e. the *z*-positions corresponding to the MN base, half-height and tip) were acquired. (Fig. 7a). In more detail, the MN base view (first row of Fig. 7c–e) shows what is happening into the collagen in correspondence of the base of the needle, while the half-height view (second row of Fig. 7c–e) shows what is happening into the collagen at half height of the MN and the tip view (third row of Fig. 7c–e) shows what is happening into the collagen in correspondence of the microneedle tip. For each *z*-position the green channel (Fig. 7c–e(i)), the merged green and transmitted light (Fig. 7c–e(ii)) channels and the orthogonal projection (Fig. 7c–e(iii)) were acquired.

After 30 min from microneedle insertion inside the in vitro dermis model, the enzyme diffused from the porous microstructure of the needle only in correspondence of the base of the MN (Fig. 7c(i)-base), but not yet from the remaining length and from the tip (Fig. 7c(i)-half height and tip). The orthogonal projection shows the triangular profiles of the MN proving that its morphology is maintained at this time point (Fig. 7c(iii)).

After 2 h, the protein started to diffuse towards the collagen of the dermis model even in correspondence of the half height of the MN (Fig. 7d(i)–base, half height and tip). The orthogonal projection still shows the triangular profiles of the MN proving that its morphology is maintained also at this time point (Fig. 7d(iii)).

Finally, 24 h after microneedle insertion inside the model, the protein diffused out from the porous microstructure of the MN along its whole length (Fig. 7e(i)). Moreover, the tip is completely dissolved as shown in the orthogonal sections (Fig. 7e(iii)) where the triangular profile of the microneedle is not visible anymore, proving that, at this time point, its architecture is completely lost.

**Fig. 8:**
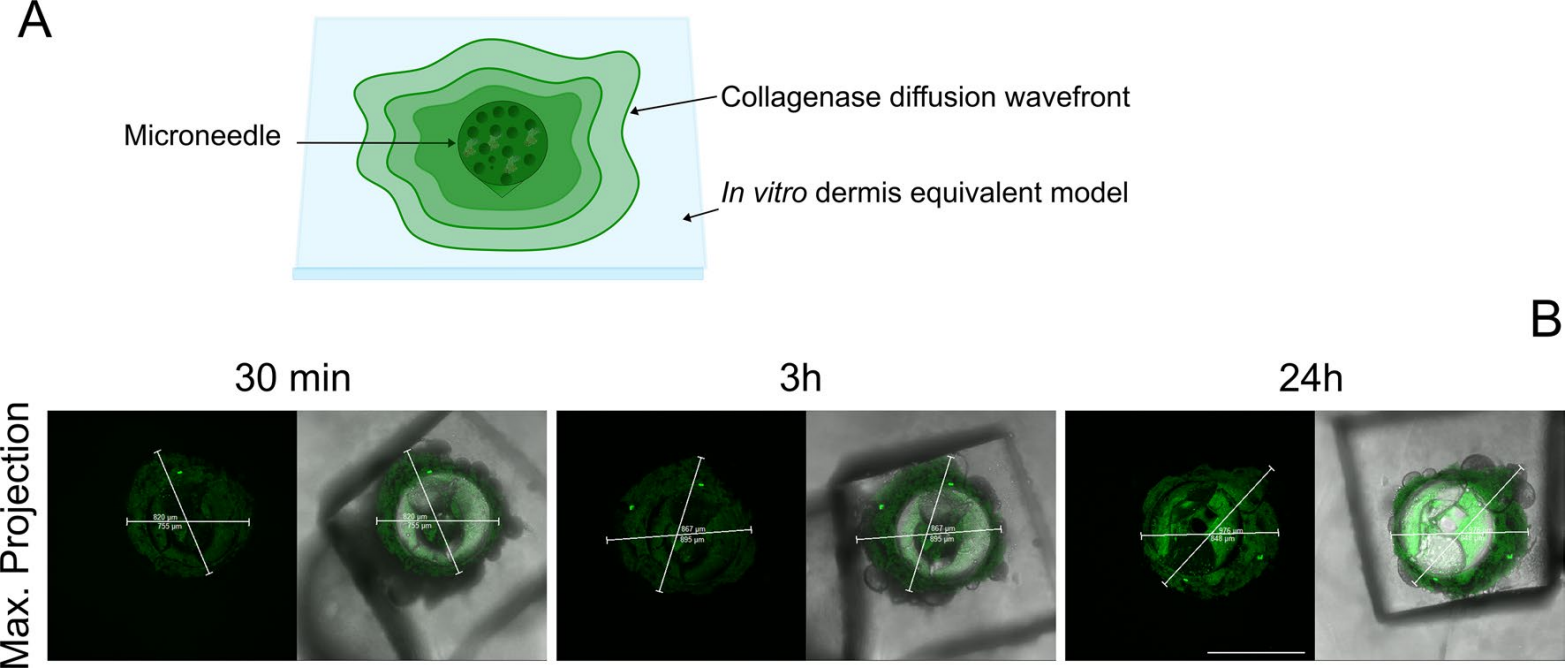
**a** Schematic representation of the collagenase diffusion wave-front. **b** Collagenase diffusion wave-front from the microneedles after 30 min, 2 h, 24 h. Scale bar 600 μm

In addition, Fig. 8 shows the maximal projection of the *z*-stack of the microneedle at the considered time points. As it can be seen from the images, the diffusion wave-front of the protein increases when increasing the release time. In more detail, after 30 min, a circular green signal of about 750 µm in diameter is visible, so the wave-front is of about 150 µm from the base. After 2 h, the wave front is of about 295 µm. Finally, after 24 h the wave-front is of about 376 µm. Moreover, especially after 24 h, the inevitable signal attenuation due to the protein diffusion and dilution into the whole collagen volume has to be taken into account when observing the images.

## Discussion

The use of simple MN based methods allows intradermal drug delivery through a minimally invasive strategy (Jamaledin et al. 2020; Park et al. 2019). Moreover, the employment of polymers which are well recognized as excipients in pharmaceutical formulations is known to preserve proteins from degradation, stabilizing the drug within the microneedle preparation (Zelikin et al. 2016).

Pharmaceutical agents can be embedded in polymeric microneedles fabricated using biodegradable polymers such as PLGA, for a sustained release, or water-soluble polymers as PVP, so that microneedles fast dissolve releasing their payload (Ita 2017).

In this work, novel biodegradable microneedles made up of PLGA together with a PVP copolymer, were designed, fabricated and analyzed to reach intermediate release rates of the encapsulated active pharmaceutical ingredient. Microneedles were produced with a stamp-less technique based on the electro-drawing principle. In a previous work on electro-drawn MNs (Vecchione et al. 2014), the electric field, able to promote the ED, was achieved by a punctual thermal stimulus to a pyroelectric crystal by means of a hot tip. Consequently, it was not possible to electro-draw microneedles in parallel and they presented a pedestal at their basis. To optimize the delivery of the drug embedded in the MN, a double dispensing procedure was implemented according to which the drug was only present in the second top drop. In a more recent work (Ruggiero et al. 2018) as well as in this paper, a novel ED setup was optimized, based on the direct pattern of titanium spiral-shaped heaters onto the pyroelectric crystal surface, to control the generated electric field at the microscale, consequently obtaining a faster fabrication of the MNs. Here, for the first time the electro-drawing was applied to mixtures of PVP/VA and PLGA with the aim of obtaining faster release profiles as compared with pure PLGA.

Both PVP/VA and PLGA are Food and Drug Administration (FDA) approved polymers for in vivo administration, widely used as drug delivery carriers for microneedles (Ke et al. 2012; Schwarz, 1990; Stevanovic et al. 2011). PVP microneedles fast dissolve after insertion within the biological tissues due to the PVP high water solubility (Sullivan et al. 2008; Sun et al. 2013; Thakur et al. 2016). Its monomer *N*-vinyl pyrrolidone is able to copolymerize with acrylic acid, methacrylates and other vinyl monomers and the formed polymers are widely used in biomedical applications such as contact lenses (Lai 1997), fast and controlled delivery of drugs (Yaung and Kwei 1998), and for immobilization of the enzymes (Basri et al. 1999). Recently many swelling studies of PVP based hydrogels have been published, in which PVP has been blended with pectin to improve its mechanical properties (Bajpai 2000; Maolin et al. 2000; Mishra et al. 2008; Park and Nho 2003).

On the contrary, PLGA-based devices (Cabezas et al. 2014; Klose et al. 2008), as well as PLGA-based MNs (Li et al. 2019), require long time from several days to weeks to release drugs and thus are usually employed for sustained release. In particular, the drug release rates are influenced by factors such as polymer molecular weight, poly lactic acid (PLA)/poly glycolic acid (PGA) ratio (Makadia and Siegel 2011), the geometry of the devices, the drug type, and drug release medium (Klose et al. 2008). All these factors need to be considered to have the desired drug release mechanism. In detail, the PLGA degradation can be accelerated by increasing PLGA hydrophilicity and the chemical interactions among the hydrolytic groups or reducing the volume to surface ratio of the device (Makadia and Siegel 2011). For example, for drug releases up to one month, a polymer with high hydrophilicity is recommended, while for a longer-term release of several months, a PLGA with high molecular weight could be suitable (Houchin and Topp 2009). Furthermore, it has been well established that PLGA can encapsulate small molecules, peptides or proteins and distribute them with different periods of time and routes of delivery (Battisti et al. 2019; Fischer et al. 2009; Kim et al. 2013a, b).

To promote faster release rates, one strategy is to obtain porosity in PLGA matrices. For example porogen agents showed being a simple method and have been widely employed to realize pores (Clark et al. 2014; Ni et al. 2017; Ullah et al. 2017; Xu et al. 2017). Recently, trehalose was used as a porogen for the release of hydrophobic drugs from PLGA-based MNs (Zhao et al. 2020).

In our case, with the aim of increasing PLGA porosity, we fabricated microneedles starting from water-in-oil (W/O) emulsions containing lecithin, which is a surfactant, and maltose as emulsion stabilizer. Maltose was chosen because, being emulsions instable systems, they have the tendency of undergoing a phase-separation before consolidation (i.e. before starting the MN fabrication), especially for high concentrations of the dispersed phase. Recently, in the case of W/O emulsions, the addition of maltose in the water phase has been proposed as an effective way to enhance stability (Esposito et al. 2016). On the other hand, maltose has been previously introduced in literature as effective material for the fabrication of dissolving drug delivery systems, such as microneedles (Lee et al. 2011).

The consolidation of the porous microstructure occurred through solvent evaporation from the continuous phase of the W/O emulsion. The sole PLGA in the oil phase, together with lecithin and maltose in the water phase, brought to the formation of big pores, but such pores had a closed morphology. The addition of PVP/VA in the oil phase allowed to obtain microneedles with an interconnected porosity, where the polymer chains of PVP, being PVP hydrophilic, were entrapped together with the drug (Browne et al. 2020). These microneedle microstructure and porosity are related to the amount of drug that can be loaded inside the polymeric matrix and to the MN release rate. The introduction of PVP/VA allowed the formation of an interconnected porosity, increasing the protein release to the 27% in 3 h compared to the 6.5% of the sole PLGA.

Another method to control and eventually increase the release rates of pharmaceutical components embedded in PLGA matrix is to use PLGA microparticles (MPs) delivered by microneedle patches. In this way the release is characterized by an initial burst followed by a slight linear phase (Battisti et al. 2019; Mazzara et al. 2019). Even if it is not excluded that MPs could be introduced to fabricate multi-compartmental microneedles by electro-drawing, one of the advantages of producing MNs starting from W/O emulsions is that the process is one step and thus very fast.

Moreover, a main advantage is that our process showed to work well with proteins without destroying their activity. Indeed, the ability to release drugs in a timeframe of few hours is compatible with labile molecules such as proteins like collagenase which otherwise, for longer release times, can start to degrade due to the wet environment surrounding the delivery system. It is reported that when the enzyme is in contact with water, for example in the case of reconstitution for injectable formulations, it has to be injected within 8 h to avoid degradation problems (Vaccaro et al. 2017). At the same time, this time-frame is advantageous as compared to an immediate release where protein is immediately exposed to the degrading biologic environment and part of it may be degraded before acting (Chi et al. 2003; Volkin and Klibanov 1989; Zhu et al. 2000). In addition, maintaining high systemic but non-toxic drug exposures may be useful to preserve a sufficient molecule activity in vivo, when degradation mechanisms start (Lai and Crews 2017; Shire et al. 2004).

For this reason, we think that the obtained materials could be used for the delivery of a broader spectrum of labile molecules such as antibodies, peptides or small biomolecules.

## Conclusion

This work presents fast degradable porous microneedles obtained with a stamp-less electro-drawing based approach. Electro-drawing is a fast, mold-free, one-step, mild temperature and easily tunable drug delivery strategy that overcomes the technological limitations of both micromolding and drawing lithography. However, the formulations used so far in ED microneedles are PLGA-based with long release times, which are not compatible with labile biomolecules. In this work, ED microneedles were drawn starting from water-in-oil (W/O) emulsion drops and a drug with full-blown dermo-cosmetic activities, such as collagenase, was embedded in the microcavities created within the polymeric phase. In this regard, being the inner microneedle structure related to the drug release, we studied different possible water-in-oil precursor formulations to tune pore morphology and collagenase release profiles to obtain protein releases in an active form and in a time-frame of few hours. This was possible by replacing PLGA with a polymeric matrix made of poly(lactic-*co*-glycolic acid)(PLGA) with Poly(1-vinylpyrrolidone-*co*-vinyl acetate) (PVP/VA) to accelerate degradation and using a water-in-oil emulsion to provide a porous matrix. The resulting microneedles were able to pierce the stratum corneum of cadaver porcine skin and collagenase was able to diffuse in a controlled manner over the time in a 3D in vitro dermis model.

These findings could have significant implications for the development of new cosmetic applications as well as for skin disease therapies.

## Electronic supplementary material

Below is the link to the electronic supplementary material.

Supplementary file1 (DOCX 26232 kb)
