# Supplementary material for: Engineered PLGA-PVP/VA based formulations to produce electro-drawn fast biodegradable microneedles for labile biomolecule delivery
Source: Prog Biomater. 2020 Nov 3;9:203–17. doi: 10.1007/s40204-020-00143-2 (PMC7718351; doi:10.1007/s40204-020-00143-2)
Supplement: Supplementary file 1 — Supplementary file1 (DOCX 26232 kb) [file 40204_2020_143_MOESM1_ESM.docx]

**Engineered PLGA-PVP/VA based formulations to produce electro-drawn fast biodegradable microneedles for labile biomolecule delivery**

**Supplementary Materials**

Valentina Onesto***^a*^***, Concetta Di Natale***^a,b^***, Martina Profeta***^a^***, Paolo Antonio Netti***^a,b^***, Raffaele Vecchione***^a,b,*^***

***^a^****Center for Advanced Biomaterials for HealthCare@CRIB, Istituto Italiano di Tecnologia, Largo Barsanti e Matteucci 53, Napoli 80125, Italy*

***^b^****Interdisciplinary Research Center of Biomaterials, CRIB, University Federico II, P.leTecchio 80, Naples 80125, Italy*

*Correspondence should be addressed to:

Dr. Valentina Onesto

Center for Advanced Biomaterials for HealthCare@CRIB, Istituto Italiano di Tecnologia, Largo Barsanti e Matteucci 53, Napoli 80125, Italy

e-mail: [valentina.onesto@iit.it](mailto:valentina.onesto@iit.it)

Dr. Raffaele Vecchione

Center for Advanced Biomaterials for HealthCare@CRIB, Istituto Italiano di Tecnologia, Largo Barsanti e Matteucci 53, Napoli 80125, Italy

Tel: +39 081 19933100

Fax +39 081 19933140

e-mail: [raffaele.vecchione@iit.it](mailto:raffaele.vecchione@iit.it)

**Fig. S1** Collagenase-ATTO 488 calibration curve in DMSO/SDS/NaOH.

**Fig. S2** Collagenase-ATTO 488 calibration curve in TRIS buffer pH 7.1.


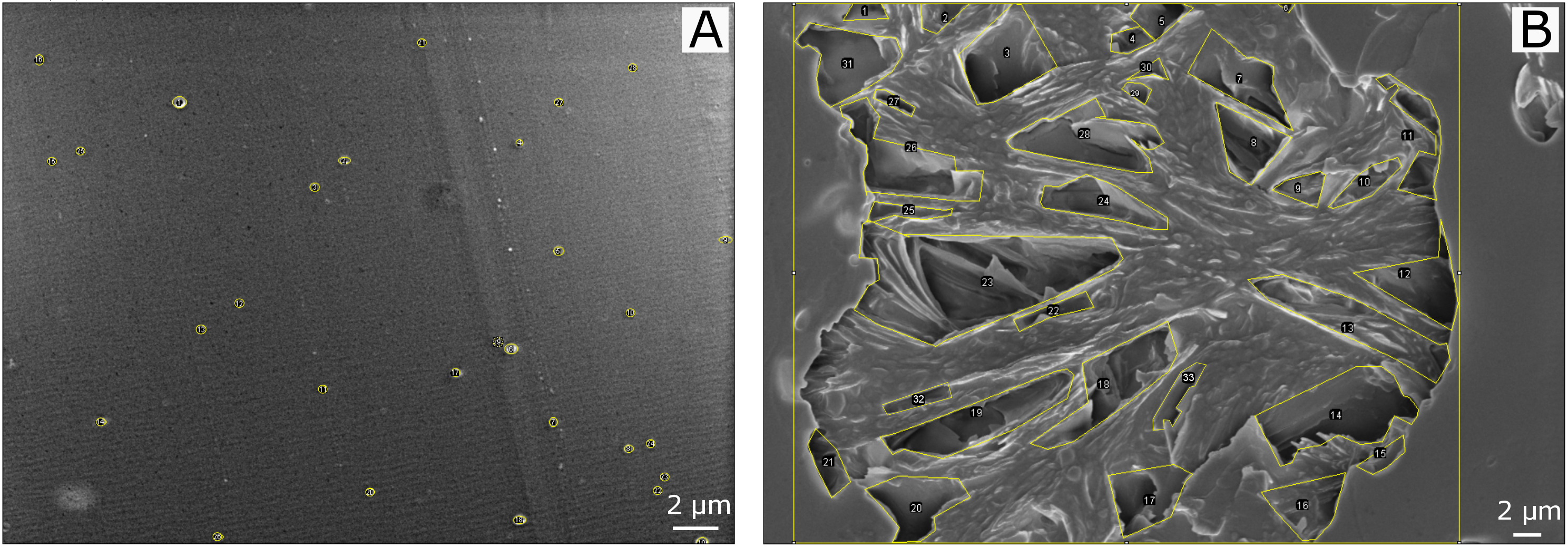


**Fig. S3** Examples of regions of interest (ROIs) on SEM images of MNs fabricated starting from (A) PLGA with ammonium bicarbonate and (B) PVP/VA+PLGA with lecithin and maltose emulsions. From these ROIs pore sizes and porosity percentages were extracted.


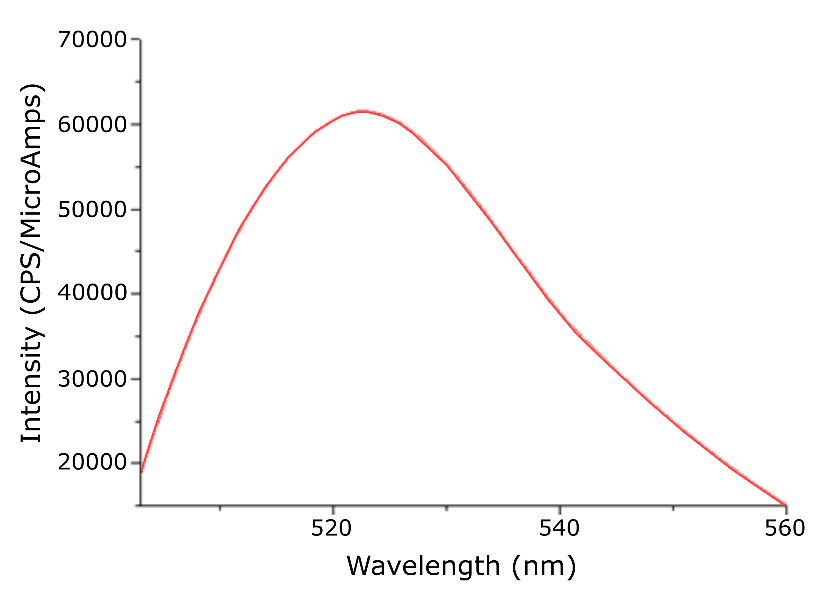


**Fig. S4** Experimental quantification by fluorescence of Collagenase loading in 300 µm electro-drawn microneedles.


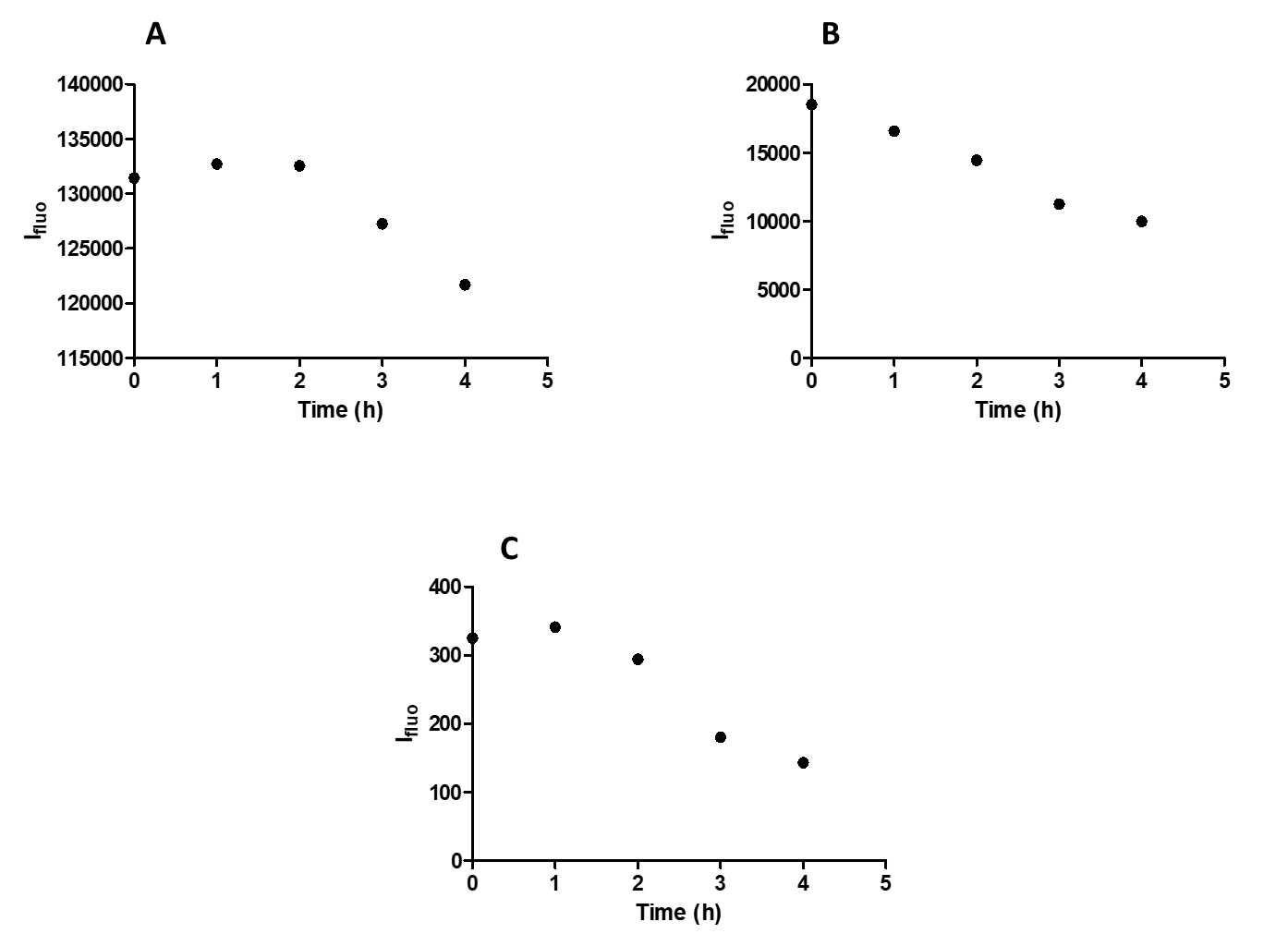
**Fig. S5** Fluorescence analysis of collagenase at 37°C in TRIS buffer pH 7.1 at three different concentrations A) 22µg/mL, B) 0.22 µg/mL and C) 0.022 µg/mL and for different time periods (0-4 h)


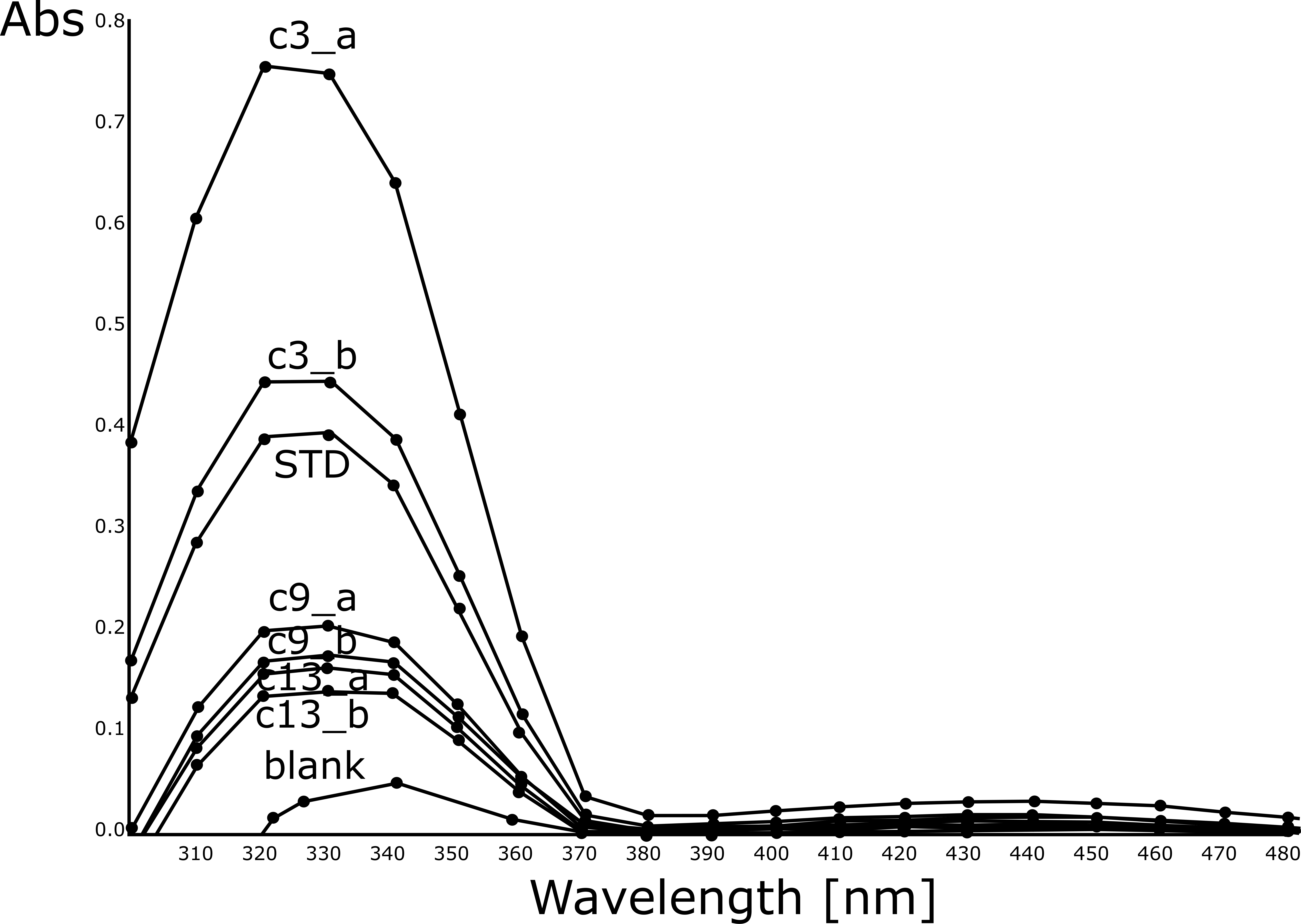


**Fig. S6** Collagenase activity in the three samples analyzed (in duplicate). Sample 1 is PLGA+AB, 2 PLGA+PVP/VA+LM, 3 PVP/VA+LM. The blank is the polymer emulsion without collagenase.
